# Associations between social relationships and cognitive impairment in older adults: A systematic review of risk and protective factors

**DOI:** 10.1097/MD.0000000000047753

**Published:** 2026-03-06

**Authors:** Jie Li, Leiyu Shi, Nan Jiang, Lixue Wang, Yujie Wang, Zhuozhao Zheng

**Affiliations:** a Department of Health Policy and Management, Bloomberg School of Public Health, Johns Hopkins University, Baltimore, MD; b Department of Radiology, Beijing Tsinghua Changgung Hospital, School of Clinical Medicine, Tsinghua Medicine, Tsinghua University, Beijing, China; c School of Healthcare Management, Tsinghua Medicine, Tsinghua University, Beijing, China; d Department of Social Services, Beijing Tsinghua Changgung Hospital, Tsinghua University, Beijing, China; e Medical Data Science Center, Beijing Tsinghua Changgung Hospital, School of Clinical Medicine, Tsinghua Medicine, Tsinghua University, Beijing, China.

**Keywords:** cognitive impairment, meta-analysis, protective factors, risk factors, social networks, social relationships

## Abstract

**Background:**

This study aims to systematically review the association between social relationships and cognitive impairment in the elderly, and to identify risk and protective factors through quantitative meta-analysis, providing evidence to inform cognitive health intervention strategies.

**Methods:**

PubMed, Embase, Web of Science, CNKI, and other databases were systematically searched for studies published before November 1, 2024. Eligible studies included cross-sectional and cohort studies involving adults aged ≥60 years. Separate meta-analyses were conducted for ratio-based effect sizes (OR/HR/RR) and regression coefficients (β) to synthesize the associations between social relationships and cognitive impairment.

**Results:**

From 1013 retrieved records, 11 studies were included. Meta-analysis showed that reduced social networks and social isolation were significantly associated with an increased risk of cognitive impairment (e.g., loneliness and mild cognitive impairment: OR = 2.89, 95% CI = 1.19–7.02). In contrast, larger social networks and frequent social participation were associated with a substantially lower risk of cognitive impairment, with participation in ≥1 social activity linked to an approximately 64% risk reduction. β-based meta-analysis further indicated positive associations between social support and cognitive performance, while specific activities such as volunteering conferred additional protective effects. Moderate heterogeneity was observed across social relationship dimensions.

**Conclusion:**

Social relationships play an important role in maintaining cognitive health in older adults. This meta-analysis provides quantitative evidence supporting both the risk and protective effects of social relationships on cognitive impairment, underscoring the importance of promoting social engagement to support cognitive health in aging populations.

## 1. Introduction

Cognitive impairment is a common health problem among older adults, characterized by a persistent decline in memory, thinking, and cognitive abilities.^[1,2]^ This situation not only seriously affects the quality of life and independence of the elderly, but also places a heavy economic burden on families and society.^[3]^ According to statistics, there are approximately 50 million patients with cognitive impairment worldwide, with the elderly accounting for the highest proportion, and approximately 10 million new cases occur every year.^[4,5]^ It is expected that by 2050, the number of patients with cognitive impairment will exceed 150 million, and the incidence is particularly significant in low- and middle-income countries.^[6,7]^ For example, the prevalence of cognitive impairment among older adults in China is close to 15%, a trend that demonstrates the significant public health significance of this issue.^[8]^ Therefore, it is crucial to identify the influencing factors of cognitive impairment and develop effective interventions. In recent years, social relationships have received widespread attention as an important factor influencing cognitive health.^[9,10]^ Research shows that social support, the size of social networks, and the frequency of social participation may contribute to slower cognitive decline, potentially through mechanisms such as psychological stimulation, emotional support, and cognitive reserve. However, research in this area has yet to reach consistent conclusions.^[11,12]^

Although there have been preliminary studies exploring the association between social relationships and cognitive impairment, the results have been highly variable.^[13,14]^ Some studies suggest that richer social relationships may effectively delay cognitive decline, whereas others fail to identify a clear or stable association.^[15]^ These discrepancies may partly arise from substantial heterogeneity in how social relationships are defined and measured across studies, which limits the comparability of findings and complicates interpretation.^[16,17]^

In addition, existing research has not fully clarified which specific dimensions of social relationships function as risk factors or protective factors for cognitive impairment. Many studies focus primarily on structural indicators such as social network size, while relatively fewer have systematically examined functional aspects, including the quality of social support or the frequency and type of social engagement.^[18]^ This imbalance restricts a comprehensive understanding of the mechanisms through which social relationships influence cognitive health.

Furthermore, evidence derived from different cultural and socioeconomic contexts remains fragmented. While several cross-sectional and cohort studies have reported that frequent social participation is associated with a lower risk of cognitive impairment,^[19–22]^ other studies have found only cross-sectional associations without longitudinal causal effects.^[23–25]^ Variability in study design, outcome measures, and sociocultural background may contribute to these inconsistencies.^[26–28]^ Consequently, a systematic and quantitative synthesis is needed to clarify the overall association between social relationships and cognitive impairment and to identify key risk and protective factors across diverse populations.^[23]^

Other studies have tried to analyze the impact of specific social activities (such as volunteering activities, interacting with friends) on specific cognitive dimensions, but there is still a lack of sufficient mechanism exploration.^[29,30]^ Overall, although the current study has initially revealed the potential association between social relationships and cognitive impairment, there are still significant deficiencies in the uniformity of research methods and indicators, cross-cultural applicability, and analysis of causal mechanisms.^[23]^

Based on the above background and issues, this study aims to comprehensively evaluate the association between social relationships and cognitive impairment in the elderly through a systematic review and meta-analysis, clarify the risk and protective factors in social relationships, and explore their effects across different cultural contexts. The research results will provide scientific basis for intervention strategies to improve the cognitive health of the elderly and point out the direction for further research in related fields.

## 2. Materials and methods

### 2.1. Literature search

A comprehensive literature search was conducted in multiple electronic databases, including PubMed, Embase, Web of Science, and CNKI, to identify relevant studies published up to November 1, 2024. The search strategy was developed using a combination of controlled vocabulary terms and free-text keywords related to cognitive impairment and social relationships.

For English-language databases, the following search string was applied:

(“cognitive impairment” OR cognition OR “cognitive reserve”) AND (“social relationships” OR “social relations” OR “social networks” OR “social support” OR “social participation” OR “social isolation”) AND (elderly OR “older adults” OR aging).

For Chinese-language databases, equivalent Chinese terms were used, including “认知障碍,” “认知功能,” “社会关系,” “社会网络,” “社会支持,” “社会参与,” “社会隔离,” and “老年人.”

The Boolean operators AND/OR were applied consistently, and duplicate or redundant terms were removed to ensure a clear and reproducible search strategy. In addition, reference lists of the included studies were manually screened to identify any potentially relevant articles not captured by the initial database search.

### 2.2. Inclusion and exclusion criteria

Inclusion criteria: Research subjects: elderly people aged ≥60 years with cognitive impairment; Research type is cross-sectional or cohort study; Literature reports the relationship between social relationships and cognitive impairment in the elderly.

Exclusion criteria: non-original research, such as reviews, conference abstracts, etc; low-quality, repeated, incomplete or unclear research; literature does not contain the relationship between social relationships and cognitive impairment in the elderly.

### 2.3. Screening method and data extraction

The initial screening was completed by 2 independent researchers based on the title and abstract, and studies that were irrelevant or did not meet the inclusion criteria were excluded. Further screening involved full-text reading to ensure that the included literature fully met the criteria. Disagreements were resolved through discussion or third-party arbitration. For the included studies, 2 researchers independently extracted data, covering: basic information of the study, characteristics of the subjects, and outcome indicators. All extracted data were cross-validated to ensure accuracy and consistency. Inconsistencies were resolved through discussion or expert consultation.

### 2.4. Literature quality evaluation

The methodological quality of the included observational studies was independently assessed by 2 reviewers using the Newcastle–Ottawa Scale (NOS), which is specifically designed for cohort and cross-sectional studies. For cross-sectional studies, an adapted version of the NOS was applied.

The NOS evaluates study quality across 3 domains: selection of study participants, comparability of study groups based on design or analysis, and ascertainment of exposure and outcomes.

Each study was awarded a maximum of 9 points, with higher scores indicating better methodological quality. Studies were categorized as high or moderate quality according to the total NOS score (Table 2). In addition, domain-level risk-of-bias judgements (low, unclear, or high) were derived based on NOS criteria and summarized graphically. Discrepancies between reviewers were resolved through discussion or consultation with a third reviewer.

### 2.5. Statistical analysis and meta-analysis

Meta-analyses were conducted when at least 2 studies reported comparable effect estimates. Given the expected clinical and methodological diversity across observational studies, pooled analyses were performed using a random-effects model. For ratio-based effect sizes (odds ratios, hazard ratios (HRs), or relative risks), effect estimates were transformed to the natural logarithmic scale and pooled with their corresponding standard errors. For studies reporting 95% confidence intervals, standard errors were derived from the CI limits.

Because some included studies reported regression coefficients, a separate meta-analysis was performed for studies providing standardized or unstandardized regression coefficients (β). These β estimates were pooled independently from ratio-based effect sizes to avoid mixing different effect metrics.

Between-study heterogeneity was assessed using Cochran Q statistic and quantified using the *I*^2^ statistic and τ^2^. Publication bias and small-study effects were evaluated by visual inspection of funnel plots and tested using Egger regression asymmetry test when applicable. Forest plots and funnel plots were generated to present pooled estimates and assess potential publication bias. Statistical analyses were performed using appropriate meta-analysis software (e.g., R or Stata), and a 2-sided *P*-value <.05 was considered statistically significant where relevant.

## 3. Results

### 3.1. Literature search results

**Figure 1.**
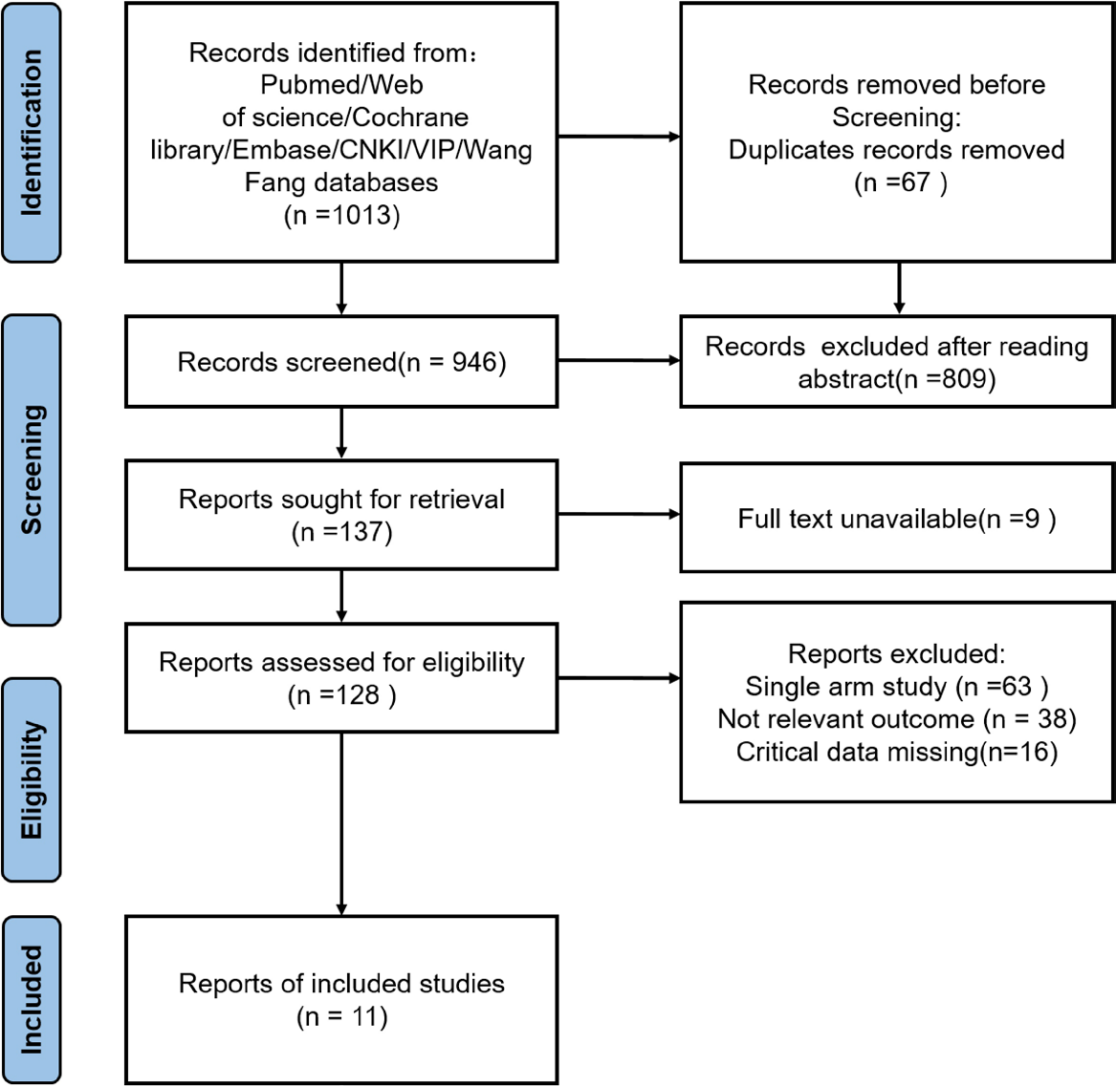
Schematic diagram of literature screening process and results.

We retrieved 1013 articles on the relationship between social relationships and cognitive impairment in the elderly from Chinese and English databases in the initial search. After removing 67 duplicate articles using literature management software, 946 articles remained. After preliminary screening, 809 articles that did not meet the inclusion criteria were excluded, and 137 articles remained. After removing 9 articles that could not obtain the full text, the full text was read and evaluated in detail, and 117 articles were further excluded. Finally, 11 studies were included in the meta-analysis, see Figure 1, and the general information of the included literature is shown in Table 1.

**Table 1:** Basic characteristics of included literature.

No.	First author (Year)	Country	Study design	Follow-up	Sample size (N)	Age (yr)	Social factor assessed	Cognitive outcome	Measurement tools	Effect size
1	Foong (2021)	Malaysia	Cross-sectional	NA	308	≥60	Social networks (LSNS-6)	Global cognitive function (continuous)	MMSE	β
2	Dodds (2024)	Australia	Cross-sectional	NA	201	≥65	Social networks (contact frequency, network size)	Global cognitive function (continuous)	MoCA	β
3	Lee (2024)	South Korea	Prospective cohort	14 years	2673	≥65	Social frailty (multi-domain)	Incident cognitive impairment (binary)	KLoSA cognitive battery	HR
4	Ma (2024)	China	Prospective cohort	NA	3803	≥65	Social support and cognitive activity	Incident cognitive impairment (binary)	MMSE	OR
5	Trujillo Tanner (2023)	USA	Prospective cohort	NA	1197	≥60	Social isolation	Global cognitive function (continuous)	Neuropsychological battery	β
6	Yin (2020)	China	Prospective cohort	NA	6586	≥65	Social support (family/children contact)	Incident cognitive impairment (binary)	MMSE	OR
7	Brenowitz (2014)	USA	Prospective cohort	NA	8953	≥65	Social relationships (marital status, living arrangement, social contact)	Incident mild cognitive impairment (binary)	Uniform MCI diagnostic criteria	HR
8	Putra (2025)	Indonesia	Cross-sectional	NA	1024	≥60	Social participation	Cognitive impairment (binary)	IFLS cognitive test	OR
9	Li (2019)	China	Cross-sectional	NA	3418	≥60	Social networks and community engagement	Cognitive impairment (binary)	MMSE	OR
10	Fu (2018)	China	Cross-sectional	NA	8966	≥60	Social activity frequency	Global cognitive function (continuous)	CHARLS cognitive measures	β
11	Bian (2023)	China	Cross-sectional	NA	720	≥65	Social support (SSRS score)	Cognitive impairment (binary)	Computerized cognitive test	OR

Effect size: OR; HR; β, regression coefficient.

HR = hazard ratio, KLoSA = Korean Longitudinal Study of Aging, LSNS-6 = Lubben Social Network Scale–6, MMSE = Mini-Mental State Examination, MoCA = Montreal Cognitive Assessment, OR = odds ratio, IFLS = Indonesia Family Life Survey, SSRS = social support rating scale, MCI = mild cognitive impairment, NA = not applicable.

### 3.2. Literature quality evaluation

**Figure 2.**
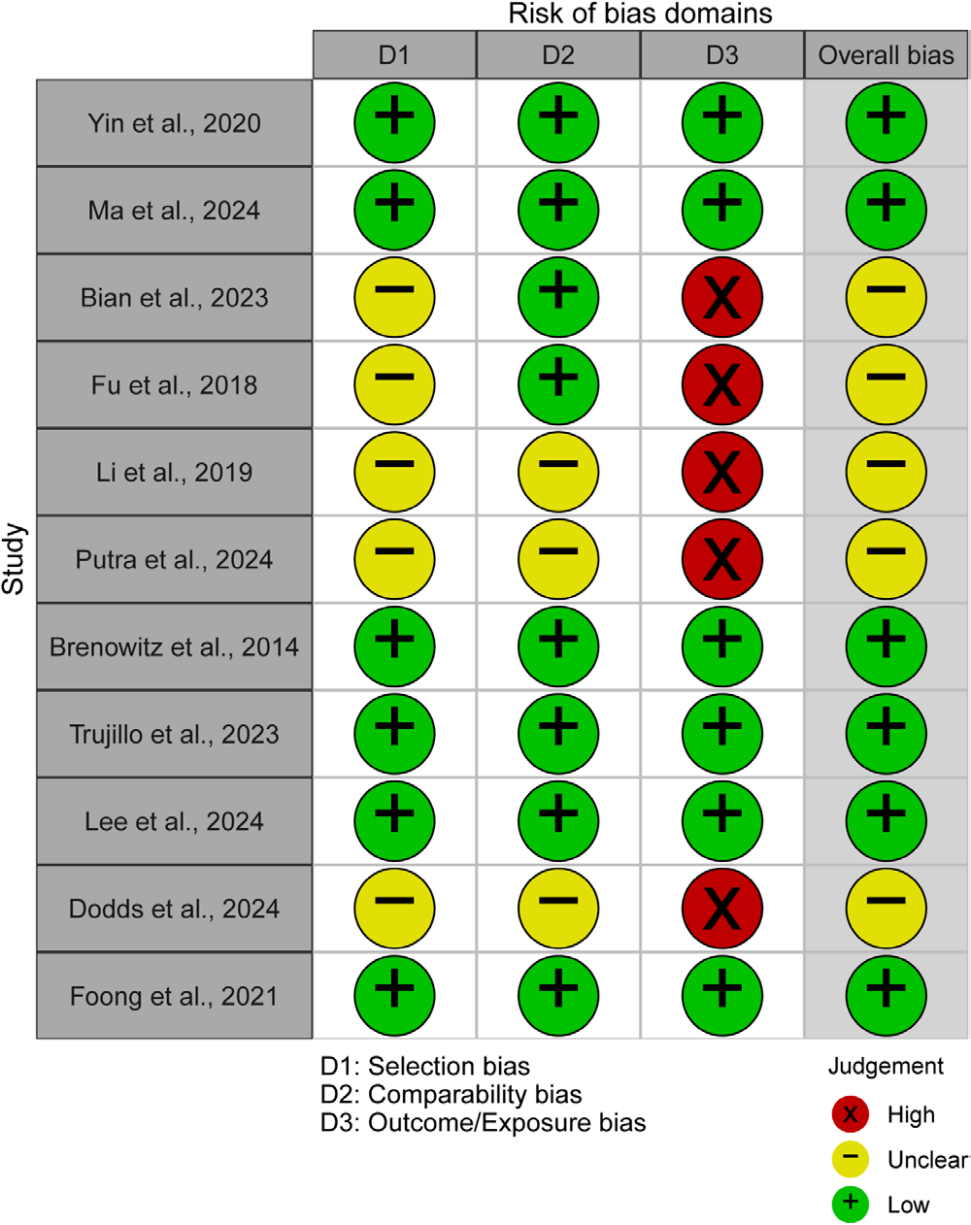
Domain-level risk-of-bias assessment of included studies. Each study was evaluated across 3 domains: selection domain (D1), comparability domain (D2), and outcome/exposure domain (D3). Judgements are displayed using a traffic-light system, where green circles indicate low risk of bias, yellow circles indicate unclear risk, and red circles indicate high risk of bias. An overall risk-of-bias judgement is also presented for each study. Risk-of-bias judgements were derived from domain-level assessments based on the Newcastle–Ottawa Scale (NOS) criteria.

**Figure 3.**
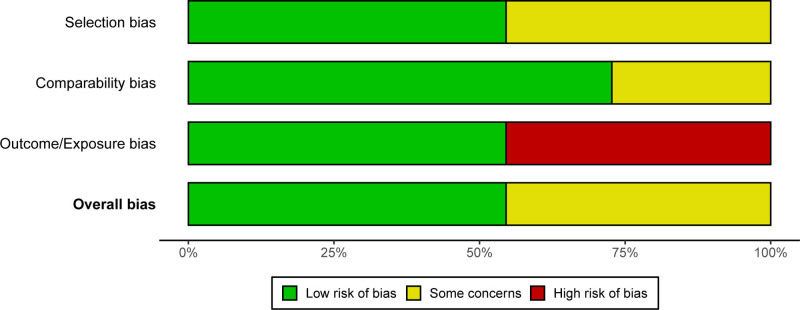
Summary of domain-level risk-of-bias judgements across included studies. The figure shows the proportion of studies classified as having low risk of bias, some concerns (unclear risk), or high risk of bias for each assessment domain and the overall judgement, based on domain-level evaluations. Colors correspond to the level of risk of bias, with green indicating low risk, yellow indicating some concerns, and red indicating high risk.

Based on the NOS assessment, the included studies demonstrated overall moderate to high methodological quality. Most studies achieved satisfactory scores in participant selection and outcome assessment, while comparability across study groups varied depending on the extent of covariate adjustment. In addition, domain-based risk-of-bias judgements indicated that no study was classified as having a critical overall risk of bias. The overall quality of the included evidence was therefore considered acceptable for synthesis (Figs. 2 and 3). Detailed NOS-based methodological quality scores for each included study are provided in Table 2.

**Table 2:** Methodological quality assessment of included studies using the NOS.

PMID	Study Design	Selection (0–4)	Comparability (0–2)	Outcome/Exposure (0–3)	Total (0–9)	Quality
32317993	Prospective cohort	4	2	3	9	High
38191348	Prospective cohort	4	2	3	9	High
37754212	Cross-sectional	3	2	1	6	Moderate
29385773	Cross-sectional	3	2	1	6	Moderate
31608097	Cross-sectional	3	1	1	5	Moderate
38769637	Cross-sectional	3	1	1	5	Moderate
24577205	Prospective cohort	4	2	3	9	High
37568997	Prospective cohort	4	2	3	9	High
38968954	Retrospective cohort	4	2	3	9	High
38438951	Cross-sectional	3	1	1	5	Moderate
34876024	Prospective cohort	4	2	3	9	High

NOS = Newcastle–Ottawa Scale.

### 3.3. Meta-analysis results

#### 3.3.1. Meta-analysis of ratio-based effect sizes (OR/HR/RR)

A total of 6 studies reporting ratio-based effect sizes, including odds ratios, HRs, or relative risks, were included in the meta-analysis. A random-effects model was applied to account for potential heterogeneity across studies. The pooled results suggested that lower levels of social participation or social support were associated with an increased risk of cognitive impairment, whereas higher levels of social engagement were associated with a reduced risk.

**Figure 4.**
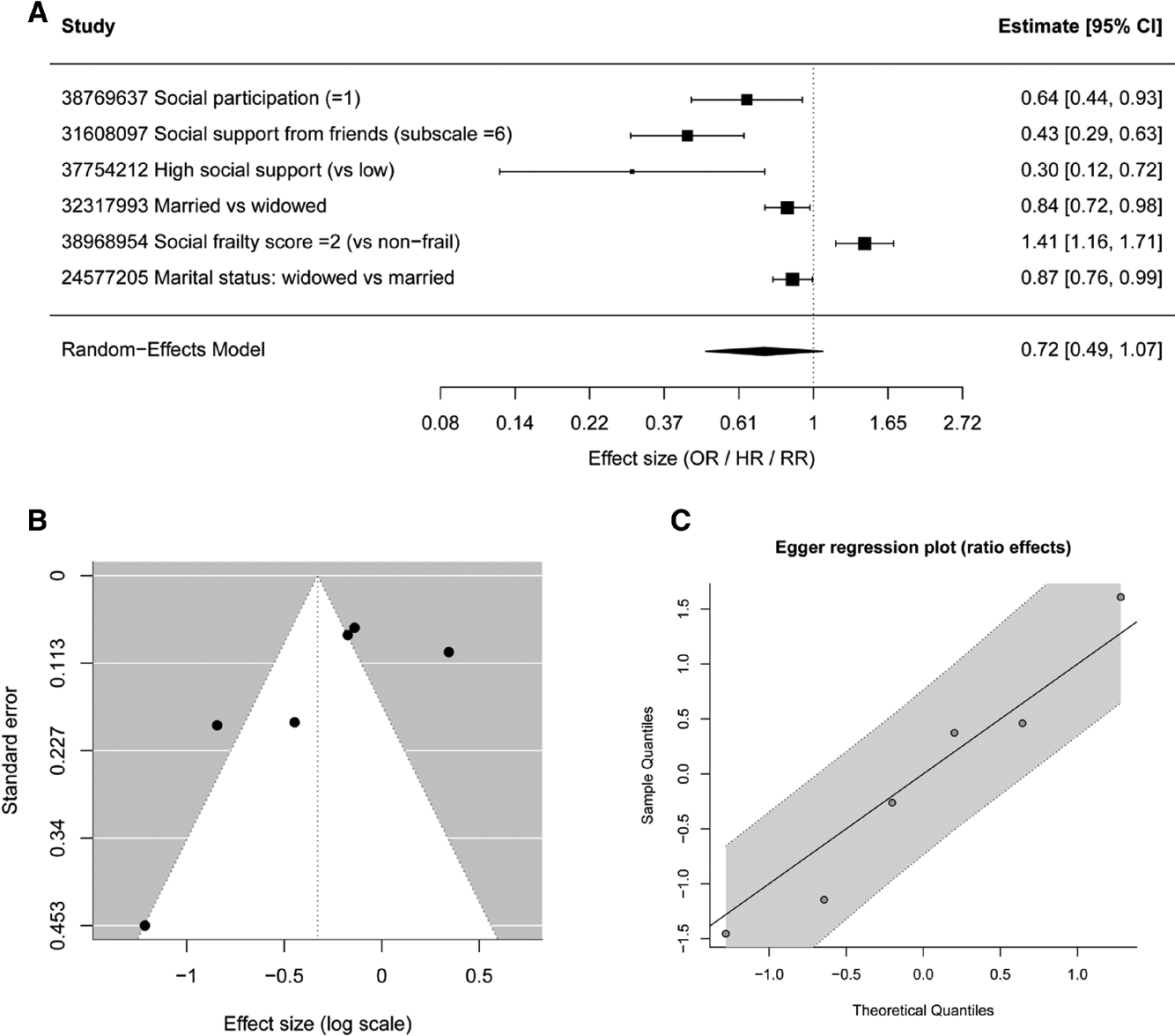
Meta-analysis of ratio-based effect sizes (OR/HR/RR) for the association between social relationships and cognitive impairment. (A) Forest plot showing individual study estimates and the pooled effect size derived from a random-effects model. Effect sizes are presented as odds ratios, hazard ratios, or relative risks with corresponding 95% confidence intervals. (B) Funnel plot used to visually assess potential publication bias among the included studies. (C) Egger regression plot evaluating small-study effects for ratio-based effect sizes.

The individual study estimates and the overall pooled effect are presented in the forest plot (Fig. 4A). Overall heterogeneity was low, indicating reasonable consistency across studies. Visual inspection of the funnel plot did not reveal substantial asymmetry (Fig. 4B). In addition, Egger regression test showed no significant evidence of small-study effects, suggesting the absence of publication bias among the included studies (Fig. 4C).

#### 3.3.2. Meta-analysis of regression coefficients (β)

Four studies reporting standardized or unstandardized regression coefficients (β) were included in a separate meta-analysis. A random-effects model was used to pool the regression coefficients. The combined results indicated that higher levels of social support or stronger social networks were positively associated with cognitive performance, whereas social isolation or social frailty was associated with poorer cognitive outcomes.

**Figure 5.**
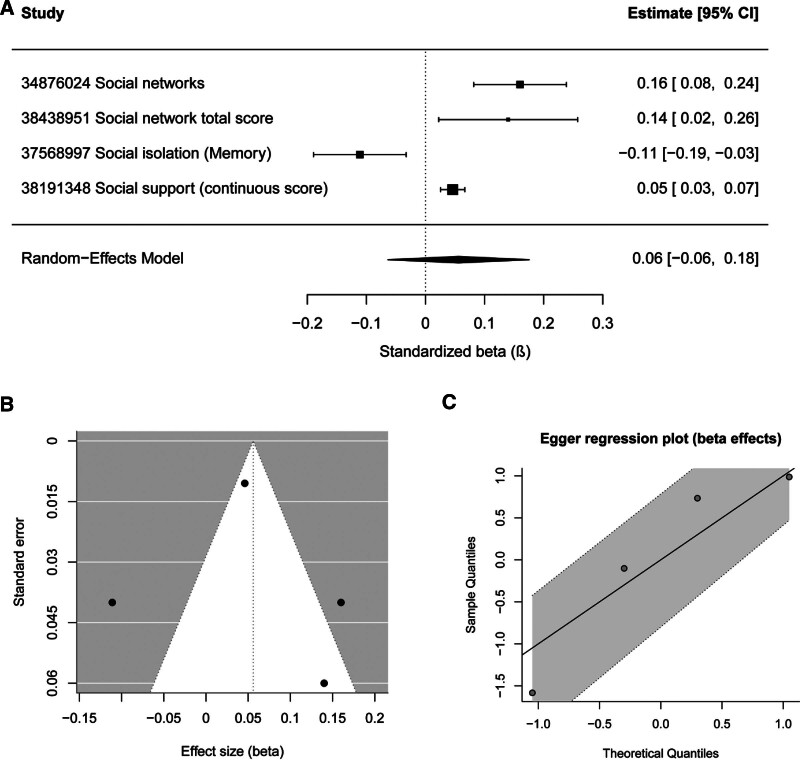
Meta-analysis of regression coefficients (β) for the association between social relationships and cognitive outcomes. (A) Forest plot displaying study-specific regression coefficients (standardized or unstandardized β) and the pooled estimate obtained using a random-effects model, with 95% confidence intervals. (B) Funnel plot assessing potential publication bias for β-based effect sizes. (C) Egger regression plot examining the presence of small-study effects in the β-based meta-analysis.

The pooled regression coefficients and study-specific estimates are shown in the forest plot (Fig. 5A). Funnel plot inspection demonstrated no clear asymmetry (Fig. 5B). Consistently, Egger regression analysis did not indicate significant small-study effects, further supporting the absence of publication bias in the β-based meta-analysis (Fig. 5C).

## 4. Discussion

### 4.1. Overview of the association between social relationships and cognitive impairment

Based on the quantitative synthesis presented in the meta-analysis, our findings provide robust evidence that social relationships are significantly associated with cognitive impairment in older adults, functioning as both risk and protective factors depending on their structure and quality. Cognitive function includes aspects such as perception, memory, imagination, and thinking. It is a multidimensional cognitive process influenced by genetic inheritance, lifestyle, and psychosocial factors. Among these, social participation is considered an important determinant of cognitive function in older adults. Two main theoretical frameworks have been proposed to explain the association between social participation and cognitive function. The exhaustion and disuse theory suggests that reduced social participation may accelerate cognitive decline through insufficient cognitive stimulation, whereas sustained mental and physical engagement may help preserve cognitive function. Accordingly, engagement in intellectual and physical activities has been associated with a lower risk of cognitive decline in later life. The social identity theory posits that social participation reinforces individuals’ sense of group membership, which may influence values, emotional well-being, and health-related behaviors, thereby indirectly supporting cognitive health through enhanced social resources and emotional support.

### 4.2. Social relationships and cognitive impairment

All 11 articles studied the correlation between various aspects of social relationships, including social connections, social networks, social isolation, and cognitive impairment. Foong et al study showed that the elderly with smaller social networks had the lowest level of cognitive function.^[31]^ Dodds et al study also showed the same result.^[32]^ Those residents with larger social networks of family and friends had significantly better cognitive performance. However, Yorgason et al study studied the impact of social networks on cognition and instrumental activities of daily living with an average interval of 10.9 years.^[33]^ Social isolation was cross-sectionally associated with cognitive function, and there was no evidence of a longitudinal association between social networks and cognitive impairment.

### 4.3. Dimensional effects of social relationships

Social relationships play an important role in maintaining cognitive functions and preventing cognitive impairment, which are mainly reflected in the following dimensions.^[34,35]^ First, social network size is closely related to cognitive function. Research shows that having a larger social network is associated with higher cognitive function scores, particularly in executive function and language abilities.^[36,37]^ Conversely, smaller social networks may exacerbate both physical and cognitive deterioration, especially among older adults.^[38]^ Second, the frequency of social interactions is also an important factor affecting cognitive health. Individuals who interacted regularly with family and friends (e.g., at least twice a week) had a significantly lower risk of cognitive impairment.^[14,39]^ Frequent interaction can enhance psychological flexibility and cognitive reserve, thereby delaying the decline of cognitive function to a certain extent.^[40,41]^ Furthermore, the quality of social support has a positive effect on cognitive health. High-quality emotional support and practical assistance can effectively relieve the psychological stress and anxiety of the elderly, thus exerting a protective effect on cognitive function.^[42,43]^ However, research has also found that excessive social support may weaken an individual’s autonomy, thereby adversely affecting mental health. Participating in different types of social activities, such as religious activities and volunteering, also has significant benefits for cognitive health. Diverse social activities not only provide individuals with cognitive stimulation but also reduce the risk of cognitive impairment by enhancing social interaction.^[16,44]^ Finally, different cultural and geographical backgrounds have different effects on social relationships. For example, in Asian cultures, family support contributes more significantly to cognitive health, whereas in Western societies, community and friend support play a greater role in cognitive health.

### 4.4. Risk and protective factors

Social support has a positive effect on cognitive function in the elderly and buffers cognitive decline. These findings emphasize the importance of promoting face-to-face and remote social participation opportunities to improve the well-being of the elderly in different cognitive function subgroups, and shows that social support has a positive effect on some executive functions in Africa. Putra et al study showed that social isolation and loneliness (3%) increased the odds of MCI/dementia (OR = 2.89, 95% CI = 1.19–7.02). Cognitive impairment was also associated with older age (≥75 years), unmarried status, female sex, rural residence, and lack of social participation. After controlling for other variables, elderly people with lower education who participated in at least 1 social activity in the past 12 months had a 0.64-fold lower risk than those who did not participate in social activities. Hwang study showed that greater social isolation was associated with increased anxiety in patients with cognitive impairment (coefficient = 0.7242, t = 2.51, *P* = .015). Based on this, Brenowitz et al investigated the specific risk of the relationship between social relationships and cognitive impairment in the elderly. The results showed that compared with married participants, widowed participants had a significantly lower risk of cognitive impairment (HR: 0.87; 95% CI: 0.76–0.99), but participants who were divorced/separated or never married did not. The risk of cognitive impairment was significantly higher when living with others than when living with a spouse/partner (HR: 1.35; 95% CI: 1.03–1.77), and the risk of mild cognitive impairment was not related to having children or siblings. These results did not consistently identify social relationships as a strong risk factor for mild cognitive impairment, or an independent clinical predictor.

### 4.5. Effects of different social activities on specific cognitive dimensions

Dodds et al studied the cross-sectional relationship between social network characteristics, cognition, quality of life, and frequency and type of service activities in the previous month for the elderly in 2 long-term care environments, indicating that people with larger social networks, including connections between family and friends, have better cognitive abilities. The relationship between social networks and cognitive function was independent of quality of life, specific activity types, and attendance. The results of the Ko study showed that the elderly who participated in social activities less than once were more likely to experience cognitive impairment and cognitive deficits than those who participated in social activities 4 or more times.^[45]^ This is consistent with previous research results that lack of social activities in the elderly is a risk factor for cognitive decline, physical frailty, and cognitive impairment. The factor that reduces the risk of cognitive impairment in elderly people with low education is participation in one or more social activities (*P* = .019; AOR0; *P* = .003; 0.744; 95% CI, 0.574–0.962). In summary, social interaction-based social participation is beneficial in delaying cognitive impairment in the elderly.

### 4.6. Potential biological and psychological mechanisms

Social relationships play a key role in the occurrence and development of cognitive impairment, and their underlying mechanisms can be examined from both biological and psychological perspectives.^[46,47]^ Understanding these mechanisms not only provides a theoretical basis for the observed associations but also informs the development of targeted intervention strategies.

At the biological level, social relationships influence cognitive function through several interrelated pathways.^[48,49]^ Accumulating evidence from psychoneuroimmunology and social neuroscience indicates that social experiences can exert measurable effects on immune and neurobiological functioning.^[50]^ Positive social relationships have been associated with lower levels of chronic systemic inflammation, potentially through reduced release of pro-inflammatory markers such as C-reactive protein and interleukin-6,^[51]^ which are known contributors to accelerated cognitive decline.^[52]^ A comprehensive synthesis by Slavich and Irwin demonstrated that adverse social experiences activate inflammatory signaling pathways via neural–immune interactions, providing a biologically plausible link between social stress and cognitive outcomes.^[50]^ In addition, sustained social interaction and cognitive engagement may promote neuroplasticity by strengthening neuronal connectivity and enhancing cognitive reserve, particularly in brain regions critical for memory and executive function, such as the hippocampus and prefrontal cortex.^[53,54]^ Evidence from neuroimaging and experimental studies further suggests that socially enriched environments can facilitate synaptic plasticity and neural resilience, thereby buffering age-related cognitive decline.^[55]^ Social relationships may also modulate hypothalamic–pituitary–adrenal axis activity by alleviating chronic stress, thereby reducing cortisol-related hippocampal damage and slowing cognitive decline.^[56,57]^ Such stress-buffering effects of social support have been consistently observed in both human and animal studies, supporting a neuroendocrine pathway linking social relationships to cognitive health.^[58]^

At a psychological level, social relationships have a positive effect on cognitive function by shaping emotional well-being and health-related behaviors.^[59]^ Emotional support derived from social relationships can alleviate loneliness and anxiety, psychological states that have been linked to impairments in attention and memory and may accelerate cognitive decline.^[23]^ Moreover, social connectedness may enhance psychological resilience and indirectly promote cognitive health by encouraging engagement in health-promoting behaviors, including physical activity and cognitively stimulating activities.^[60]^ These psychological pathways may operate synergistically with biological mechanisms, jointly contributing to the protective effects of social relationships on cognitive function in later life.

### 4.7. Summary and implications

Overall, this systematic review and meta-analysis demonstrated a significant association between social relationships and cognitive impairment in older adults. The findings indicate that social network size, social isolation, and loneliness exert substantial influences on cognitive function, whereas active engagement in social activities is associated with a reduced risk of cognitive impairment. In particular, frequent participation in social activities and involvement in specific forms of social engagement, such as volunteering, appear to confer protective effects against cognitive decline, including more severe cognitive impairment.

By synthesizing and extending previous evidence, the present study provides a more comprehensive understanding of the relationship between social relationships and cognitive impairment. Consistent with earlier studies by Foong et al and Ko et al,^[45,61]^ our findings support the view that limited social networks and social isolation are important risk factors for cognitive impairment. Importantly, the meta-analytic results further highlight the long-term protective role of sustained social participation, suggesting that psychological stimulation and social support derived from social activities may play a critical role in delaying cognitive decline. In addition, this study expands existing knowledge by demonstrating that the adverse effects of social isolation may be more pronounced among older adults with lower educational attainment or those residing in rural areas, which aligns with prior evidence emphasizing the moderating influence of socioeconomic factors on cognitive health.^[62]^ Moreover, different types of social activities were found to exert differential effects on cognitive outcomes, with social participation activities showing particular relevance for cognitive preservation. These observations are consistent with social cognitive theory and suggest that social engagement may promote cognitive health through both psychological and biological pathways, such as stress reduction, attenuation of chronic inflammation, and enhancement of neural connectivity.^[63,64]^

The findings of this study also have important implications for interventions aimed at promoting cognitive health in older populations. Enhancing opportunities for social participation at both community and family levels may represent a feasible and effective strategy to mitigate cognitive decline. Community-based programs, including interest groups, volunteer services, and social gatherings, may facilitate social interaction among older adults, while family support can provide essential emotional and interpersonal engagement. For individuals with limited mobility or geographic constraints, digital communication technologies, such as video calls and online social platforms, may offer alternative avenues to reduce social isolation and maintain social connections. Collectively, these approaches have the potential not only to delay cognitive decline but also to improve overall quality of life in later life.

Despite the strengths of this study, several limitations should be acknowledged. First, there was substantial heterogeneity in how social relationships were defined and measured across the included studies, encompassing diverse structural and functional indicators such as social network size, social participation frequency, and perceived social support. This variability may have influenced the comparability of effect estimates and contributed to between-study heterogeneity. Second, the evidence synthesized in this meta-analysis was derived exclusively from observational studies, including cross-sectional and cohort designs, which limits the ability to establish causal relationships between social relationships and cognitive impairment and raises the possibility of residual confounding. Third, cognitive impairment was assessed using different instruments across studies and cultural contexts, including variations in screening tools, diagnostic thresholds, and normative standards. Such cultural and methodological differences in cognitive assessment may affect the consistency and generalizability of the pooled results. Finally, although formal assessments of publication bias did not indicate significant small-study effects, the possibility of publication bias cannot be entirely excluded, particularly given the limited number of studies included in some meta-analyses and the tendency for studies with null findings to remain unpublished. Future research incorporating standardized measures, culturally sensitive cognitive assessments, and randomized or quasi-experimental designs is warranted to strengthen causal inference and enhance the robustness of conclusions.

## 5. Conclusion

This study confirmed the risk and protective effects of social relationships on the cognitive health of the elderly through a systematic review and meta-analysis. The results showed that smaller social networks and social isolation significantly increased the risk of cognitive impairment, while frequent social activities had a significant protective effect. This finding provides a scientific basis for designing intervention strategies to optimize cognitive health in the elderly, emphasizing that by promoting social participation and improving social support may help delay cognitive decline and improve quality of life.

## Author contributions

**Conceptualization:** Jie Li, Leiyu Shi, Zhuozhao Zheng.

**Data curation:** Jie Li, Nan Jiang, Lixue Wang.

**Formal analysis:** Nan Jiang, Yujie Wang.

**Methodology:** Jie Li, Leiyu Shi.

**Project administration:** Yujie Wang, Zhuozhao Zheng.

**Supervision:** Leiyu Shi, Zhuozhao Zheng.

**Validation:** Lixue Wang.

**Visualization:** Yujie Wang.

**Writing – original draft:** Jie Li.

**Writing – review & editing:** Leiyu Shi, Lixue Wang, Zhuozhao Zheng.
